# Value of novel thrombotic markers for predicting occurrence of the malignant cerebral artery infarction: a prospective clinical study

**DOI:** 10.3389/fneur.2023.1238742

**Published:** 2023-11-30

**Authors:** Xiaoxia Zhao, Siyu Yang, Ruining Lei, Yi Liu, Qiaoyan Duan, Jundong Li, Lei Sun

**Affiliations:** ^1^Department of Neurology, Shanxi Provincial Peoples Hospital, Taiyuan, China; ^2^Department of Neurology, Fifth Hospital of Shanxi Medical University, Taiyuan, China; ^3^Clinical Laboratory, Shanxi Provincial Peoples Hospital, Taiyuan, China; ^4^Medical Imaging Department, Shanxi Provincial Peoples Hospital, Taiyuan, China; ^5^Zhao Furun Famous Doctor Studio in Shanxi Province, Taiyuan, China

**Keywords:** thrombin–antithrombin complex, plasmin–α2 plasmin inhibitor complex, tissue prothrombin activator–prothrombin activator inhibitor complex, thrombomodulin, malignant cerebral artery infarction, projection

## Abstract

**Objective:**

This study investigated the diagnostic performance of thrombin–antithrombin complex (TAT), plasmin–α2 plasmin inhibitor complex (PIC), tissue plasminogen activator–plasminogen activator inhibitor complex (t-PAIC), and thrombomodulin (TM) in predicting the progression of massive cerebral infarction to the malignant cerebral artery infarction.

**Method:**

A total of 71 patients with massive cerebral infarction confirmed by imaging examination were divided into malignant cerebral artery infarction group (MCAI) and non-malignant cerebral artery infarction group (NMCAI) based on whether they progressed to MCAI after admission. TAT, PIC, t-PAIC, and TM were measured immediately after admission. The predictive performance was analyzed by the receiver characteristic operating curve (ROC).

**Result:**

The median plasma concentrations of TM, PIC, TAT, and t-PAIC in the MCAI patients at admission were 10.65 IU/mL, 1.17 μg/mL, 12.25 ng/mL, and 13.85 ng/mL, respectively, which were higher than those in the NMCAI patients (9.00 IU/mL, 1.07 μg/mL, 4.60 ng/mL, and 8.70 ng/mL), and the difference was statistically significant (*p* = 0.045, *p* = 0.035, *p* = 0.004, and *p* = 0.003). Elevated plasma t-PAIC concentration was shown to be an independent risk factor for progression of massive cerebral infarction to MCAI (OR = 1.131) by multivariate logistic regression analysis. ROC curve analysis showed that t-PAIC was the best predictor of MCAI (AUC = 74.7%), with a sensitivity of 75.0% and specificity of 75.9% when t-PAIC concentration was ≥12.4 ng/mL; TAT had the highest specificity in predicting MCAI, with a specificity of 90.7% when the TAT concentration was ≥13.5 ng/mL.

**Conclusion:**

The detection of PIC, TAT, t-PAIC, and TM is a comprehensive assessment of vascular endothelial damage and activation of the coagulation and fibrinolytic systems and has predictive value for poor prognosis in patients with MCAI. The widespread use of these tests will likely greatly improve the early diagnosis rate of MCAI.

## 1 Introduction

Large cerebral infarction in the cerebral hemisphere is a type of ischemic stroke with high mortality and disability, and its poor prognosis is often associated with the development of malignant cerebral edema. When a large cerebral infarction occurs following partial or complete occlusion of the middle cerebral artery, the secondary malignant cerebral edema leads to increased intracranial pressure and midline deviation of the brain tissue, causing deterioration of the patient’s neurological function, decreased level of consciousness, and even occurrence of brain herniation, which is called malignant cerebral artery infarction (MCAI) (1). Until recently, neuroimaging is still the prime tool for diagnosing malignant cerebral infarction. Unfortunately, it is often too late for the patients who already have severe neurological deficits or even life-threatening conditions when intracranial changes are detected on imaging. Therefore, early prediction of massive cerebral infarction in the patients prone to progress to MCAI becomes particularly important. The current research on the prevention and treatment of MCAI is significantly lagging behind, and screening out appropriate patients for early prediction and precise intervention before the onset of the disease is the key to saving lives and improving prognosis. Moreover, it would be more cost-effective and valuable than the management in the intensive care unit and surgical treatment after the onset of MCAI.

Previous studies have suggested that cerebral infarct volume is a good predictor of MCAI (2). However, its sensitivity has yet to be improved, and repeated imaging is clearly not suitable for patients with severe stroke, so the possibility of bedside markers as early warning indicators of MCAI has become a hot topic of research in recent years. The thrombin–antithrombin complex (TAT) is considered to be a sensitive marker of thrombin generation (3), plasmin–α2-plasmin inhibitor complex (PIC) is a marker of the initiation of the fibrinolytic system (4), tissue plasminogen activator–plasminogen activator inhibitor complex (t-PAIC) can be used to reflect fibrinolytic system damage (5), and endothelial cell damage is indicated by thrombomodulin (TM) (6). The only high-sensitivity chemiluminescence platform currently available internationally allows for rapid and accurate measurement and comprehensive assessment of vascular endothelial damage and activation of the coagulation and fibrinolytic systems, and thus a prognostic assessment of whether higher concentrations of novel thrombotic markers in the blood are associated with progression to MCAI in massive cerebral infarction.

## 2 Methods

### 2.1 Patients

We had taken detailed history and performed comprehensive physical examinations on patients with acute cerebral infarction admitted to the intensive care unit of our neurology department from July 2019 to March 2022. The study protocol was approved by the Human Ethics Review Committee of Shanxi Provincial People’s Hospital.

The inclusion criteria were as follows: (1) age ≥ 18 years; (2) admission within 24 h after the onset of acute cerebral infarction; (3) head CT or diffusion-weighted imaging (DWI) show Alberta Stroke Program Early CT Score (ASPECTS) < 6 (7), infarct volume ≥ 70 mL (8), or infarct area ≥ 2/3 of the middle cerebral artery blood supply area (9); (4) informed consent.

Exclusion criteria: (1) the presence of a history of traumatic brain injury within 1 week; (2) discharge or transfer to another hospital with an unknown prognosis within the observation period (7d after onset); (3) patients with a malignant tumor of any organ or system; (4) patients with injury of important organs or systems such as lung, liver, kidney, blood coagulation system, and immune system; (5) patients with severe thrombosis affecting coagulation function such as lower limb thrombosis, pulmonary embolism disease, and the use of anticoagulant drugs for injury; (6) patients with a severe neurological disability before the onset (modified Rankin Scale score mRS ≥ 2); (7) those with concomitant cerebral hemorrhage, brainstem infarction, or bilateral cerebral hemisphere infarction.

### 2.2 Baseline indicators

These include patients’ age, gender, risk factors for cerebral infarction (hypertension, diabetes, atrial fibrillation, smoking history, and alcohol drinking history), and history of whether revascularization treatment (including intravenous thrombolysis, arterial thrombectomy, and bridging treatment) performed after admission to the hospital.

### 2.3 Imaging examination

Within 24 h after the onset of the disease, head CT and/or MRI were performed to confirm massive cerebral infarction. A score of 10 for ASPECT means no early ischemic change (EIC), while a score of 0 indicates the presence of extensive ischemic foci in brain tissue. When clinical signs such as decreased level of consciousness and unequal pupil size are highly suspicious for MCAI, the head CT and/or MRI should be repeated and the presence of midline tissue displacement, ventricular, or basal pool compression should be closely observed to clarify the presence of MCAI. Two senior neuroradiologists who are unaware of the serologic test results evaluate the imaging findings separately, and a third opinion will be requested in case of disagreement.

### 2.4 Blood sampling and assay procedure

Immediately after admission to the NICU, 2.7 mL of venous blood was collected from each patient and injected into a BD vacuum blood collection tube containing 0.109 mol/L sodium citrate anticoagulant 0.3 mL (the ratio of whole blood to anticoagulant was 9:1). The above mixture was centrifuged at 3500 r/min for 15 min and take platelet-poor plasma. Eppendorf tubes were dispensed and frozen at −80°C. The assay was completed within 24 h.

TAT, TM, t-PAIC, and PIC are determined by chemiluminescent enzyme immunoassay (using the instrument: Sysmex HISCL-5000 automatic chemiluminescent analyzer and its accompanying chemiluminescent enzyme immunoassay reagents). The samples were mixed with reagents for incubation, and then, the immune reaction and chemiluminescent enzyme reaction occurred in turn. Using the high specificity of the antigen–antibody reaction and the high sensitivity of the luminescence analysis, the content of the analyte in the sample was determined rapidly and automatically by counting the photons emitted in the enzyme reaction.

### 2.5 Criteria for MCAI

Based on whether the infarction eventually progressed to malignant cerebral infarction, the groups were divided into malignant cerebral infarction and non-malignant cerebral artery infarction (NMCAI), which were determined by a senior neurologist unaware of the serologic test results. MCAI diagnostic criteria: (1) CT cross-sectional views suggesting contralateral displacement of the midline structures of the hyaline septum greater than 5 mm (10); (2) further worsening of impairment of consciousness in 1a of the NIHSS; (3) rapid neurological deterioration without other obvious causes, such as unequal or dilated fixed pupils. If all three are met, MCAI is considered to have occurred.

### 2.6 Data statistics

Normally distributed continuous variables were expressed as mean ± standard deviation (SD) and compared between groups using the *t*-test; non-normally distributed continuous variables were expressed as median (interquartile spacing) and compared between groups using the Mann–Whitney *U*-test. Categorical variables were expressed as numbers and percentages and compared between groups using chi-square tests, and Fisher’s exact probability method was used when the conditions of the chi-square test were not met. Items with a *p* ≤ 0.05 in the univariate analysis were included in the multivariate logistic regression analysis to assess the risk factors for the occurrence of malignant cerebral artery infarction. The predictive performance of different plasma marker plasma concentrations on the occurrence of MCAI was analyzed by the receiver operating characteristic curve (ROC). The Youden index was calculated; it was maximum when the best cutoff value was obtained. The area under the ROC curve (AUC) of 70–90% suggested that it was acceptable and had good accuracy. The consistency of different marker plasma concentrations in predicting MCAI was analyzed by the Hosmer–Lemeshow goodness-of-fit test. *p*-value <0.05 was statistically significant for differences. All statistical analyzes were performed using SPSS statistical software, version 25.0 (IBM Corporation, Armonk, NY).

## 3 Results

### 3.1 Baseline data analysis

From July 2019 to March 2022, 71 patients with large cerebral hemisphere infarction were enrolled in the study, 16 of whom had progression to MCAI. There were no statistically significant differences between the NMCAI group and the MCAI group in terms of gender, age, risk factors for cerebral infarction, whether or not hemodialysis was performed for hepatic function, renal function, coagulation tests, and use of antiplatelet aggregation drugs (Table 1).

**Table 1:** Comparison of baseline data between the MCAI and the NMCAI groups.

Characters		NMCAI (n = 55)	MCAI (n = 16)	χ2/ t	P-value
Gender	Male	37 (67.3%)	9 (56.3%)	0.660	0.417
	Female	18 (32.7%)	7 (43.8%)		
Age (‘X ± s years)		66.0 ± 13.0	68.9 ± 14.9	0.747	0.458
Risk factors	Hypertension	33 (60.0%)	12 (75.0%)	1.202	0.273
	Diabetes	16 (29.1%)	6 (37.5%)	0.410	0.522
	Atrial fibrillation	22 (40.0%)	6 (37.5%)	0.032	0.857
	Smoking	30 (54.5%)	7 (43.8%)	0.579	0.447
	Alcohol	28 (50.9%)	6 (37.5%)	0.893	0.345
Revascularization		21 (39.6%)	7 (46.7%)	0.239	0.625
antiplatelet therapy		19 (34.5%)	1 (6.3%)	3.606	0.058
Platelet counts (*10^9^)		200.0 (152.5,238.5)	189.0 (161.5, 251.8)	0.000	1.000
PT(s)		12.4 (11.5,13.8)	12.2 (11.8, 14.1)	0.087	0.931
APTT(s)		28.2 (25.9,30.7)	28.5 (25.1, 30.2)	0.484	0.628
Serum creatinine (umol/L)		63.8 (54.5,76.9)	63.0 (55.9, 89.1)	0.051	0.960
Urea nitrogen (mmol/L)		4.9 (4.2,6.6)	5.0 (4.1,7.4)	0.253	0.800
ALT (IU/L)		18.2 (13.3,29.3)	19.2 (14.6,28.0)	0.434	0.664
AST (IU/L)		20.9 (18.4,29.3)	31.8 (18.9,41.73)	1.735	0,083

### 3.2 Plasma concentrations of TM, PIC, TAT, and t-PAIC in different groups

**Figure 1:**
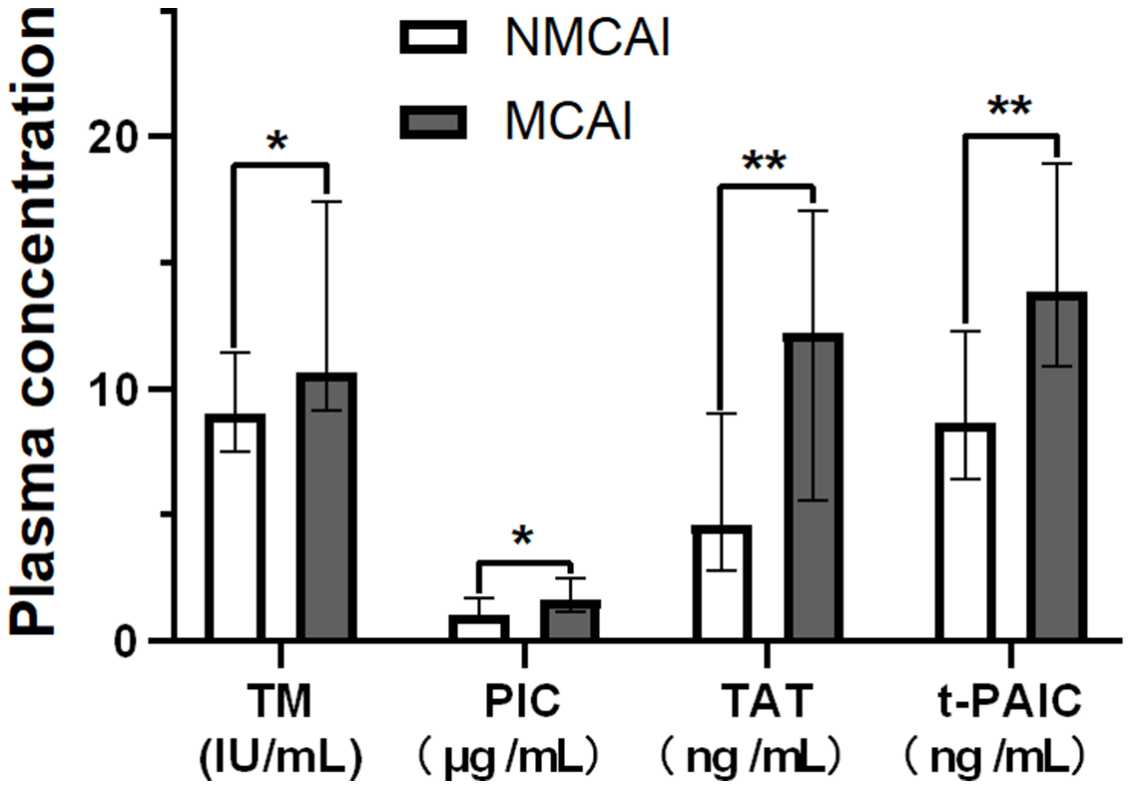
Plasma concentrations of TM, PIC, TAT, and t-PAIC in the MCI group and the NMCI group. Notice: *p<0.05, **p<0.01.

The mean plasma TM, PIC, TAT, and t-PAIC in the MCAI group were 10.65 IU/mL, 1.67 μg/mL, 12.25 ng/mL, and 13.85 ng/mL, respectively, which were higher than those in the NMCAI group (9.00 IU/mL, 1.07 μg/mL, 4.60 ng/mL, and 8.70 ng/mL). Moreover, the differences between the two groups were statistically significant (Z = -2.008, *p* = 0.045; Z = -2.106, *p* = 0.035; Z = -2.884, *p* = 0.004; Z = -2.994, *p* = 0.003; Figure 1).

### 3.3 Logistic regression analysis of risk factors for massive cerebral infarction

The differences in TM, PIC, TAT, and t-PAIC were statistically significant between the two groups, which were analyzed by multifactorial logistic regression. High plasma t-PAIC remained significant (Table 2) and was an independent risk factor for the progression to MCAI in massive cerebral infarction. For every 1 ng/mL increase in plasma t-PAIC levels in patients with massive cerebral infarction, the probability of progression to MCAI increased 1.131-fold (95% CI: 1.012, 1.264; *p* = 0.030).

**Table 2:** Logistic regression analysis of risk factors for MCAI.

Variable	OR(95%CI)	P
TM	1.158 (0.992–1.352)	0.063
PIC	0.996 (0.927–1.070)	0.909
TAT	1.070 (0.987–1.161)	0.101
t-PAIC	1.131 (1.012–1.264)	0.030

### 3.4 Analysis of the predictive performance of plasma TM, PIC, TAT, and t-PAIC concentrations on the occurrence of malignant cerebral artery infarction

**Figure 2:**
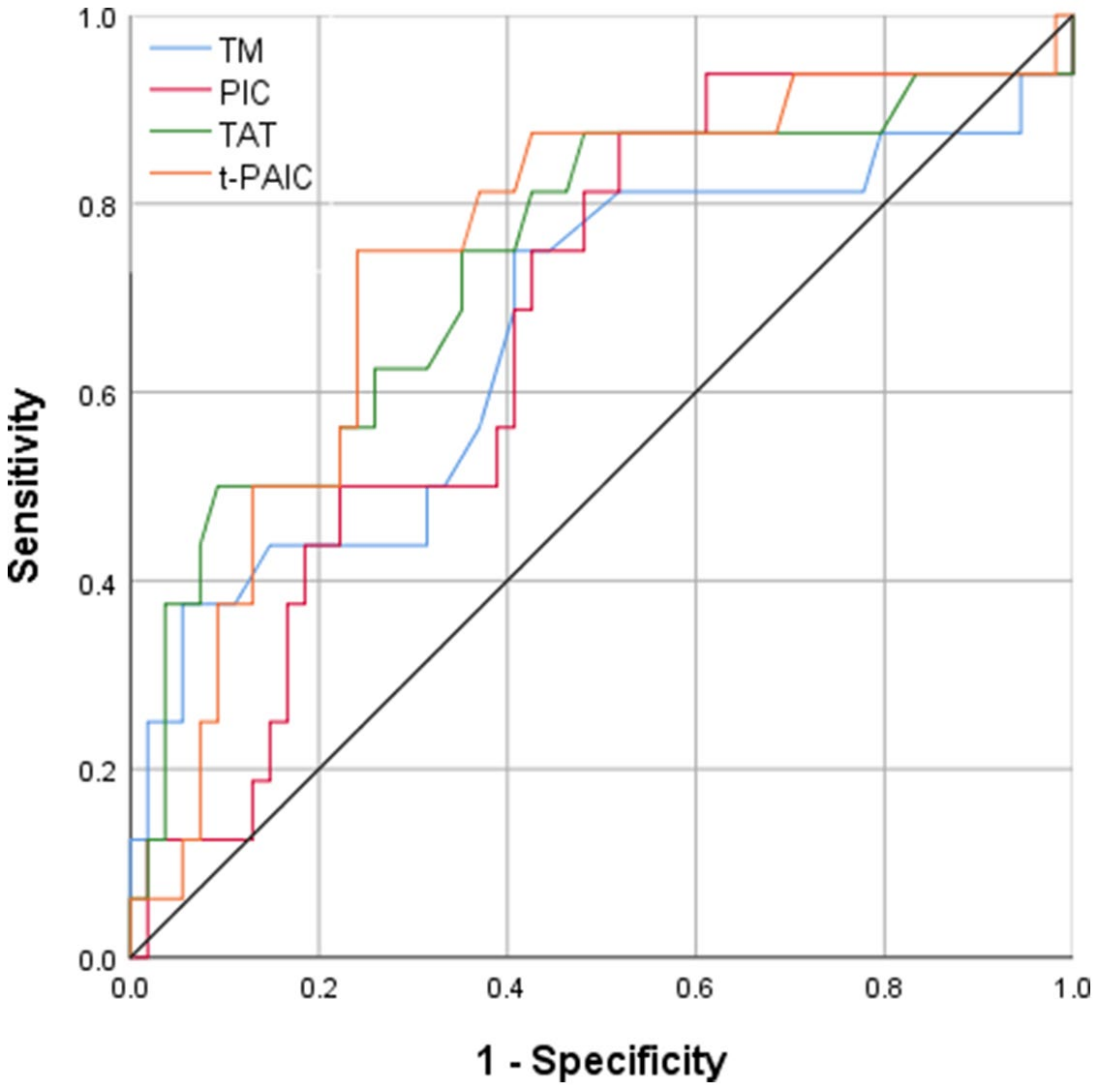
Area under ROC curve of TM PIC, TAT, and t-PAIC in patients with AIS to predict the occurrence of MCAI.

All four novel thrombotic markers showed high accuracy for the prediction of MCAI by the Hosmer–Lemeshow goodness-of-fit test analysis (Table 3). As shown by ROC curve analysis (Figure 2), t-PAIC and TAT have good predictive performance (AUC ≥ 70%), with t-PAIC showing the best predictive performance (AUC = 74.7%), higher sensitivity (75.0%), and specificity (75.9%); PIC showed higher sensitivity in predicting the occurrence of MCAI. The sensitivity was up to 87.5% when the plasma concentration of PIC was ≥1.055 μg/mL; TAT showed high specificity in predicting the occurrence of MCAI, and the specificity was up to 90.7% when the plasma concentration of TAT was ≥13.5 μg/mL.

**Table 3:** Performance analysis of four markers for predicting MCAI.

Classifier	Calibration	Discrimination	95% CI	Cutoff value	Sensitivity	Specificity
TM	80.0%	66.6%	49.6%—83.6%	9.45 IU/mL	75.0%	59.3%
PIC	77.5%	66.9%	52.3%—81.5%	1.055 μg/mL	87.5%	48.1%
TAT	77.5%	73.8%	58.5%—89.1%	13.5 ng/mL	50.0%	90.7%
t-PAIC	74.6%	74.7%	60.5%—88.9%	12.4 ng/mL	75.0%	75.9%

## 4 Discussion

The mean plasma concentrations of TM, PIC, TAT, and t-PAIC were higher in patients with malignant cerebral artery infarction than in patients with non-malignant massive cerebral artery infarction. Plasma t-PAIC was an independent risk factor for the progression of massive cerebral infarction to MCAI. Four novel thrombotic markers showed high predictive accuracy and consistency for MCAI. The discrimination of t-PAIC and TAT were both good (AUC ≥ 70%), with t-PAIC being the better (sensitivity 75.0%; specificity 75.9%); PIC sensitivity reached 87.5% when the plasma concentration of PIC was ≥1.055 μg/mL; TAT specificity could be as high as 90.7% when the plasma TAT concentration was ≥13.5 ng/mL.

TM is a transmembrane glycoprotein expressed in endothelial cells, and under physiological conditions, its plasma content is low, approximately 3.8–13.3 IU/mL. When vascular endothelial cells are injured, TM can be released from the cell membrane structure into the circulation under the action of proteases. Elevated free plasma TM content is a sensitive indicator of vascular endothelial injury (11). Plasma TM content reflects the blood–brain barrier damage to some extent. In this study, we confirmed that MCA patients who progressed to MCAI had higher plasma TM concentration (*p* = 0.045) than patients with stable MCA, which may be related to the disruption of the blood–brain barrier, the increase in permeability was irreversibly changed and gradually progressed to vasogenic edema, and the increase in tissue fluid between the cells of the brain parenchyma triggered malignant cerebral edema. The ischemic–anoxic injury occurred in the brain tissues of the central region of MCA, and the edema continued to progress beyond the physiological compensatory level leading to cranial hypertension and occupancy effects, thus further aggravating ischemia–hypoxia and edema in the swollen brain tissue in the confined cranial cavity, forming a vicious circle. Furthermore, ROC curve analysis showed that patients with massive cerebral infarction with high plasma TM levels were more likely to progress to MCAI (AUC = 66.6%).

TAT is a molecular complex composed of thrombin and thrombin inhibitors in a 1:1 ratio, and the normal plasma value of TAT is <4 ng/mL. Elevated TAT reflects the activation of the coagulation cascade reaction, indicating that thrombin is being generated and antithrombin is being continuously consumed, which is a direct evidence of enhanced coagulation activity and increased consumption of antithrombin (12). This study confirmed that plasma TAT levels were higher in patients with massive cerebral infarction who progressed to MCAI (*p* = 0.004), suggesting more thrombin production in patients with progressive disease and predicting the early hypercoagulable state of blood and thrombosis; thus, TAT can be considered as one of the predictors of progressive disease in patients with cerebral infarction. This finding is consistent with the correlation between plasma TAT concentration and disease severity found by Ye et al. (3) in related research. It has been demonstrated in animal experiments that high concentrations of thrombin-induced inflammatory cell invasion can lead to blood–brain barrier disruption and death of neural cells (13). Furthermore, ROC curve analysis showed that TAT has good predictive performance for the development of MCAI (AUC = 73.8%), with a specificity of 90.7% when plasma TAT levels are above 13.5 ng/mL, but its sensitivity is relatively low (50.0%).

t-PAIC is a complex formed by the combination of tissue-type fibrinogen activator (t-PA) and the physiological inhibitor fibrinogen activator inhibitor-1 (PAI-1). The formation of the complex inhibits the activation of fibrin by t-PA, and its high value indicates damage to the fibrinolytic system (14); simultaneous release of PAI-1 and t-PA into the bloodstream during endothelial injury can also lead to increased concentrations of t-PAI, which theoretically reflects the exogenous activation pathway of the fibrinolytic system and the damage to the vascular endothelium. In this study, we found a significant difference in t-PAI plasma levels between the MCAI and NMCAI groups (*p* = 0.003), with a greater extent of endothelial damage and poorer vascular endothelial repair capacity in patients with MCAI. Endothelial injury contributes to further thrombosis; thus, t-PAIC assists in early warning of thrombosis and indicates a more active progression of the fibrinolytic system and hemorrhagic transformation in patients with MCAI. A logistic multifactor regression analysis revealed that elevated t-PAIC plasma concentration was one of the independent risk factors for the progression of massive cerebral infarction to MCAI. ROC analysis showed that the area under the curve was 74.7%, with the best predictive performance among the four, and the sensitivity was 75.0% and the specificity was 75.9% when t-PAIC concentration was ≥12.4 ng/mL. 75.9%, suggesting being alert to the possibility of MCAI when massive cerebral infarction is accompanied by a t-PAIC level ≥ 12.4 ng/mL.

In the absence of fibronectin, plasmin which is the main enzyme that dissolves fibrin *in vivo* binds to the inhibitor α2-antiproteinase (α2-PI) in a 1:1 ratio to form a stable complex, which is the PIC (15). It has a longer half-life than that of plasmin and can be easily measured (16). Moreover, PIC does not affect the production of plasmin (17). Therefore, the plasma PIC concentration reflects the amount of fibrin produced in the blood and can be used to assess the degree of fibrinolytic activation *in vivo*, which is currently the most sensitive indicator for detecting activation of the fibrinolytic system. The normal range of plasma PIC in humans is<0.8 μg/mL. This study confirmed that PIC concentrations were elevated in patients with both MCAI and NMCAI, and the difference between the two groups was statistically significant (*p* = 0.035), suggesting patients with MCAI have a more active fibrinolytic system, a clue to be alert for hyperfibrinolysis and hemorrhagic transformation (18). The ROC analysis showed that elevated plasma PIC concentrations in patients with cerebral infarction were predictive of progression to MCAI (AUC = 66.9%), with the highest sensitivity of 87.5% among the four when the plasma PIC level was ≥1.055 μg/mL, which can be used for early assessment of the risk of progression in patients with massive cerebral infarction.

This study still has some limitations. The sample size included is small and does not fulfill the Event Per Variable requirement, so the results may not be robust enough. However, considering the rarity of this group of patients and the interpretability of the results, they are still presented. The reliability of the results needs to be confirmed by further studies.

## 5 Conclusion

The detection of the molecular markers, including t-PAIC, TAT, PIC, and TM, can be used to comprehensively assess vascular endothelial damage and activation of the coagulation as well as fibrinolytic systems. The abnormal test results show predictive value for poor prognosis in patients with massive cerebral infarction. The widespread use of these tests would be greatly improving early diagnosis of MCAI. When a patient is found to have t-PAIC ≥12.4 ng/mL, TAT ≥ 13.5 ng/mL, PIC ≥1.055 μg/mL, or (and) TM ≥ 9.45 IU/m in plasma, he/she needs to be alerted as a high-risk individual for MCAI. Decisive medical interventions or bone flap decompression to prevent the development of MCAI could be life-saving.

## Data availability statement

The raw data supporting the conclusions of this article will be made available by the authors, without undue reservation.

## Ethics statement

The studies involving humans were approved by Human Ethics Review Committee of Shanxi Provincial People’s Hospital. The studies were conducted in accordance with the local legislation and institutional requirements. The participants provided their written informed consent to participate in this study.

## Author contributions

XZ: conceptualization, project administration, methodology, and writing–review and editing. SY: investigation, data curation, formal analysis, and writing original draft. RL: writing original draft. YL: correction of manuscript. QD and JL: investigation and supervision. LS: investigation. All authors contributed to the article and approved the submitted version.
